# Research on Shipborne Helicopter Electric Rapid Secure Device: System Design, Modeling, and Simulation

**DOI:** 10.3390/s22041514

**Published:** 2022-02-15

**Authors:** Zhuxin Zhang, Qian Liu, Dingxuan Zhao, Lixin Wang, Pengcheng Yang

**Affiliations:** 1 Research Center of Special Carrier Equipment of Yanshan University, Yanshan University, Qinhuangdao 066004, China; zhzhxn@ysu.edu.cn (Z.Z.); zdx@ysu.edu.cn (D.Z.); wlx@stumail.ysu.edu.cn (L.W.); 18827551270@163.com (P.Y.); 2 Key Laboratory of Special Carrier Equipment of Hebei Province, Yanshan University, Qinhuangdao 066004, China; 3 School of Vehicle and Energy, Yanshan University, Qinhuangdao 066004, China; 4 School of Mechanical Engineering, Yanshan University, Qinhuangdao 066004, China

**Keywords:** Electric Rapid Secure Device, fuzzy control, landing assist system, power bond graph, shipborne helicopter

## Abstract

The research objective of this paper is to propose a new type of ERSD to solve the problem of the uncontrollable velocity of the claw in the current RSD. Firstly, the working characteristics of the RSD in ASIST are analyzed, and the design scheme of the transmission system of the ERSD is provided. The control system is designed by combining the vector control algorithm with the fuzzy adaptive PID control algorithm. On this basis, the trajectory planning of claw capture velocity is completed. Finally, the dynamics model of the transmission system of the ERSD is built by power bond graph theory, and the system simulation is carried out. The results show that the maximum capture time, velocity, and force were reduced by 47%, 53%, and 80%. In addition, when the ERSD is towing the helicopter, the mechanical claw can still provide good velocity tracking performance under a maximum interference load of 34,000 N.

## 1. Introduction

Many shipborne helicopters landing assist systems have been proposed to solve the problem of shipborne helicopters landing safely on medium and small ships and to improve the attendance rate of the shipborne helicopter in bad sea conditions, including Harpoon Assist Landing System, Recovery Assist Secure and Traverse System, and Aircraft Ship Integrated Secure and Traverse System (ASIST) [1,2]. ASIST, as the third generation of shipborne helicopter landing assist system, is considered a direction for future development because of its brand new landing assist idea, simplified system configuration, and superior performance indicators.

The process of the shipborne helicopter landing on the ship with the help of ASIST can be divided into the following five stages: the helicopter approaches the ship, the helicopter hovers above the deck, the helicopter lands on the deck, the helicopter is captured and secured by ASIST, and the helicopter is towed to the designated position by ASIST. Many scholars have researched the above shipborne helicopter landing process in recent years. Among them, Zan [3] and Lee et al. [4] have studied the influence of rotor thrust and fuselage load in the approach process of the shipborne helicopter by taking the Sea King helicopter as an example. Colwell [5] analyzed the influence of ships on shipborne helicopters when they hover over the deck. Wall et al. [6] further studied the dynamics of the ship-helicopter-rotor system in the hovering stage. Li et al. [7] studied the process of the shipborne helicopter colliding with the deck while landing on the ship. Zhao et al. [8] evaluated the reliability of shipborne helicopter landing ships of ASIST. Wang et al. [9,10] studied ship-helicopter system dynamics after the shipborne helicopter landed under complex sea conditions. However, few people have explored the capture and towing operation of the shipborne helicopter after landing. More specifically, few people have paid attention to ASIST’s problems of large impact force on shipborne helicopters during capture and complex towing steps during towing missions. These two problems are caused by the fact that ASIST’s execution engine, Rapid Secure Device (RSD), uses a quantitative hydraulic pump and accumulator as its power source, making the claw’s velocity uncontrollable [11,12,13].

We know that asynchronous motors have the advantages of high power density, high reliability, and high energy transmission efficiency [14,15]. Suppose the asynchronous motor is matched with a stable and reliable velocity regulation algorithm. In this case, we can use the asynchronous motor instead of the hydraulic pump and accumulator as the power source of the RSD to solve the above problems. Vector control frequency conversion technology, with its high control accuracy and good dynamic response characteristics, has become the preferred choice for the velocity regulation algorithm of asynchronous motor [16,17]. However, when the RSD is in actual operation, the axial load of the motor is constantly changing, which leads to significant changes in many parameters of the induction motor, such as resistance and inductance [18]. Moreover, the vector control algorithm and the traditional Proportion Integration Differentiation (PID) controller used with it mostly relies on the mathematical model of the motor, which makes it challenging to achieve a good velocity tracking effect in practical application [19]. With the development of intelligent control technology, many intelligent control algorithms have been designed and used for motor velocity control. The fuzzy control algorithm has become the research focus because it does not depend on an accurate model. At present, this control algorithm has been applied to permanent magnet synchronous motor and has achieved a good control effect [20,21]. If the fuzzy control algorithm and vector control algorithm are combined to design a suitable control system for the RSD, the problem of mechanical claw velocity control can be solved effectively. Liu et al. [22] proposed the realization scheme of this idea in the speed control of the asynchronous motor and proved its feasibility, which makes the above scenario possible.

In this paper, aiming at the problems existing in the capture and towing stages of the RSD, an implementation scheme of Electric Rapid Secure Device (ERSD) for shipborne helicopters is proposed for the first time. The ERSD selects an asynchronous motor as the power source and combines the vector control algorithm with the fuzzy adaptive PID (FAPID) control algorithm as a velocity tracking controller, making the velocity of the claw controllable. By planning the trajectory of the claw’s capture velocity, the ERSD ensures the capture time and minimizes the impact force. In this paper, the dynamic model of the ERSD transmission system is established based on the power bond graph theory, and the system simulation is carried out. Compared with the RSD, the ERSD proposed in this paper can significantly reduce the capture time, lessen the capture impact force, and make the traction operation easier. The ERSD expands ASIST’s range of applications to include small shipborne aircraft such as the Unmanned Aerial Vehicle (UAV) and improves the efficiency of ASIST’s towing operations.

## 2. RSD Working Characteristics

### 2.1. The Functional Principle of ASIST

**Figure 1:**
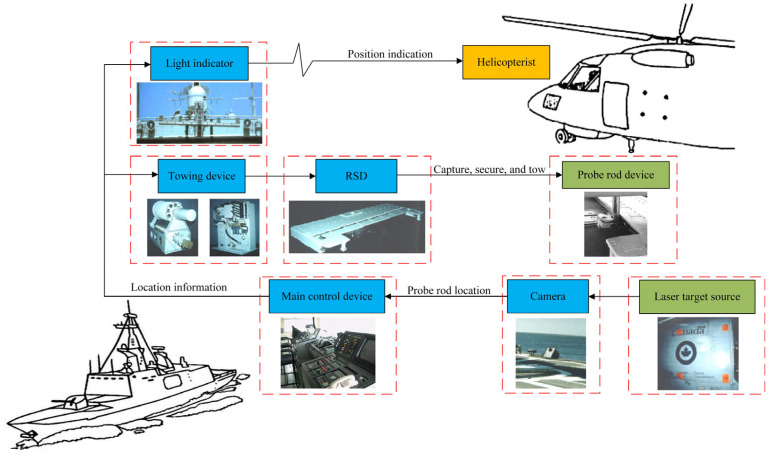
The functional principle of ASIST.

**Figure 2:**
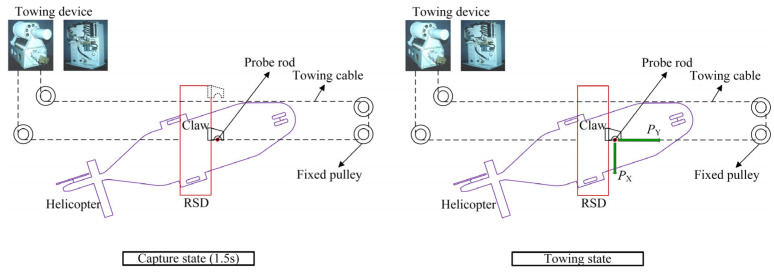
The working state of RSD and towing device.

Figure 1 shows the functional principle of ASIST. The system is mainly composed of the shipborne part and the airborne part. The shipborne part includes cameras, main control device, towing device, RSD, and light indicator, and the airborne part includes probe rod device and laser target source. The functional principle of the system is as follows:

When the shipborne helicopter reaches the upper part of the ship’s flight deck and is ready for the landing mission, the cameras installed on the deck collect the laser target source information of the helicopter. The camera calculates the helicopter’s position in real time and transmits it to the main control device. The main control device provides the helicopterist with light indication information via the light indicator to guide the helicopter to the designated landing area. At the same time, the main control device drives the RSD to track the helicopter’s position by controlling the towing device but always keeps it a certain safe distance from the helicopter. When the helicopter is in a suitable place, and the external environment allows, the helicopter begins to land. The towing device drives the RSD forward rapidly after the helicopter lands on the deck. When the RSD impacts the probe rod, the claw, mounted on the RSD, scans the capture range and captures the probe rod. Finally, the operator controls the RSD to tow the helicopter to the designated location or hangar. Figure 2 shows the working state of the helicopter’s tracking, capture, and towing by the RSD and towing device.

**Figure 3:**
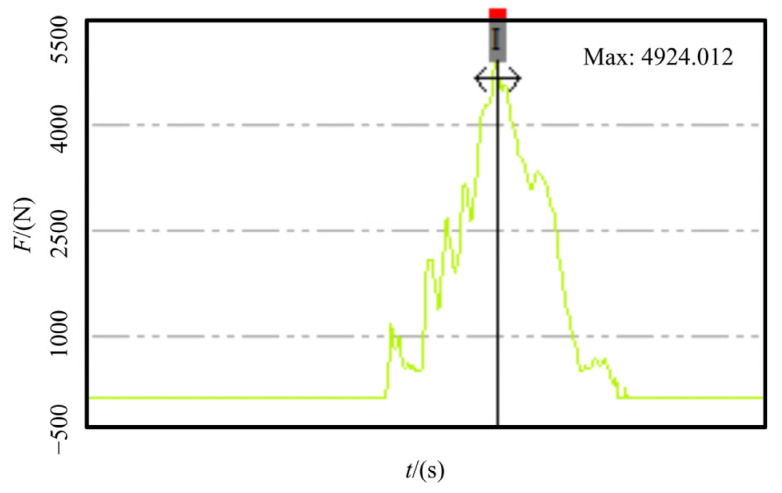
The force on the probe rod during capture.

The system can capture shipborne helicopters in up to 7-level sea conditions. The whole process from the helicopter landing to the capture does not exceed 3 s. In the second capture stage, that is, the mechanical claw of the RSD moves from the beginning to the successful capture of the probe rod; the maximum time required is not more than 1.5 s. The system also allows helicopter towing operations in up to 3-level sea conditions. However, the execution-host (RSD) of the system is limited by technical requirements and space during the design, resulting in the following two complex problems in the execution of combat tasks:

1. When performing the shipborne helicopter capture mission, the RSD uses an accumulator as the power source to drive the claw movement through a hydraulic cylinder. In this process, the velocity of the claw is uncontrollable, which leads to a significant impact force of the claw on the probe rod. As shown in Figure 3, the impact force was measured as high as 5 kN. Therefore, ASIST cannot assist the landing of small shipborne aircraft such as the UAV, which significantly limits the scope of its application;
2. When performing the shipborne helicopter towing mission, the RSD uses a quantitative hydraulic pump as its power source, making the claw’s velocity uncontrollable. As a result, the position of the shipborne helicopter needs to be adjusted repeatedly during the towing process. Moreover, the process is time-consuming, complicated, and requires highly experienced operators.

### 2.2. Force Analysis of RSD

Before the new RSD design, it is necessary to analyze the related working characteristics of RSD. It can be seen from the functional principle of ASIST that the RSD mainly undertakes the following tasks:

1. After the helicopter lands, the RSD drives the mechanical claw to capture the probe rod within no more than 1.5 s;
2. After capturing the probe rod, the RSD can reliably lock the helicopter by the claw under the condition of no more than 7-level sea conditions;
3. Under the condition of no more than 3-level sea conditions, the RSD and towing device work together to tow the helicopter to the designated position.

When the claw captures the probe rod at high velocity, the movement resistance of the claw is minimal and only comes from the inside of the RSD. When the RSD secures the helicopter or tows the helicopter, the claw bears a sizeable external load along the lateral direction.

**Figure 4:**
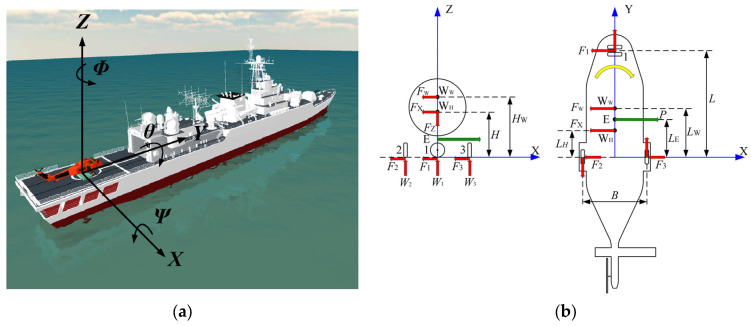
Model of helicopter and ship. (**a**) Six degrees of freedom motion of the ship; (**b**) the diagram of helicopter dynamics analysis.

Figure 4a shows the schematic diagram of the RSD working environment. Taking the center of the two rear wheels of the helicopter as the origin of the coordinates, the starboard direction of the ship as the *X*-axis forward direction, the bow direction as the *Y*-axis forward direction, and the upward direction along the plumb direction as the *Z*-axis forward direction, the spatial rectangular coordinate system is able to be established. When a ship is sailing on the sea, the flight deck will produce the following motions under the action of the ship’s power system and waves: the yaw of translation along the *X*-axis, the pitch of translation along the *Y*-axis, the heave of translation along the *Z*-axis, the pitch of rotation around the *X*-axis, the roll of rotation around the *Y*-axis, and the bow roll of rotation around the *Z*-axis.

Figure 4b shows the diagram of helicopter dynamics analysis. Assume that the helicopter’s mass is *M*_H_ and the center of mass of the helicopter is *W*_H_, which is located on the central axis of the aircraft. Assume that the distance from the *Z*-axis is *L*_H_ and the height from the deck is *H*. Assume that the force point of the claw against the probe rod is E, also located on the central axis of the aircraft, and the distance from the *Z*-axis is *L*_E_. The contact points between the three wheels and the deck are 1, 2, and 3. Assume that the distance between the centerline of the front wheelbase and rear-wheel is *L*, the distance between the two rear wheels is *B*, the maximum roll angle of the deck is *θ*, the maximum transverse wind velocity is *V*_W_, the maximum lateral acceleration is *a*_X_, and the maximum vertical acceleration is *a*_Z_.

Take the most extreme state to calculate the transverse load on the claw. Under the above rolling angle and acceleration, the thrust force of the RSD on the probe rod is *P*, and the universal axis of the front wheel is deflected 90° to the state shown in Figure 4b. Under the action of thrust *P*, the helicopter rotates in the direction of the yellow arrow. In the calculation, it can be assumed that the ship deck is tilted in the negative direction of the *X*-axis, the maximum lateral acceleration is in the positive direction of the *X*-axis, and the maximum vertical acceleration is in the positive direction of the *Z*-axis.

Assume that when the sea breeze blows in the negative direction of the *X*-axis, the wind action center is *W*_W_, located on the central axis of the aircraft, the distance from *Z*-axis is *L*_W_, and the height from the deck is *H*_W_. In the transverse direction, the windward area is *A*_W_, the drag coefficient is *C*_W_, the air density is *ρ*, and the maximum wind velocity is *V*_W_.

The calculation formula of wind force is

(1) $$F_{W}=\frac{1}{2}\rho C_{W}A_{W}V_{W}^{2},$$

where *A*_W_ × *C*_W_ = 20.

The forces acting on the center of mass *F*_X_ and *F*_Z_ are shown as follows:

(2) $$\left\{\begin{array}{l} F_{X}=M_{H}(a_{x}+g\sin\theta )\\ F_{Z}=M_{H}(a_{z}+g\cos\theta ) \end{array}\right..$$

The vertical deck force acting on the front wheel can be calculated as

(3) $$W_{1}=\frac{F_{Z}L_{H}}{L}.$$

The force balance in the *Z*-axis direction is

(4) $$F_{Z}-W_{1}-W_{2}-W_{3}=0.$$

Assume that the rolling friction coefficient of the aircraft wheel on deck is f = 0.02, and the rolling resistance of the front wheel is

(5) $$F_{1X}=W_{1}f.$$

The sum of the rolling resistance of rear wheels is

(6) $$F_{2Y}+F_{3Y}=(W_{2}+W_{3})f=(F_{Z}-W_{1})f.$$

It can be obtained by taking the moment of the *Z*-axis, which is

(7) $$P=\frac{2\left(F_{W}L_{W}+F_{X}L_{H}+F_{1X}L\right)+\left(F_{2Y}+F_{3Y}\right)B}{2L_{E}}.$$

Take a particular type of shipborne helicopter and ship as an example. Table 1 and Table 2 list the related parameters.

By substituting the data in Table 1 and Table 2 into Equation (7), it can be obtained that the thrust required by the claw in 3-level sea conditions is $P_{1}=33,121\;N$ and the securing force required by the claw in 7-level sea condition is $P_{2}=65,853\;N.$

## 3. Design of ERSD

### 3.1. Main Transmission System Design

**Figure 5:**
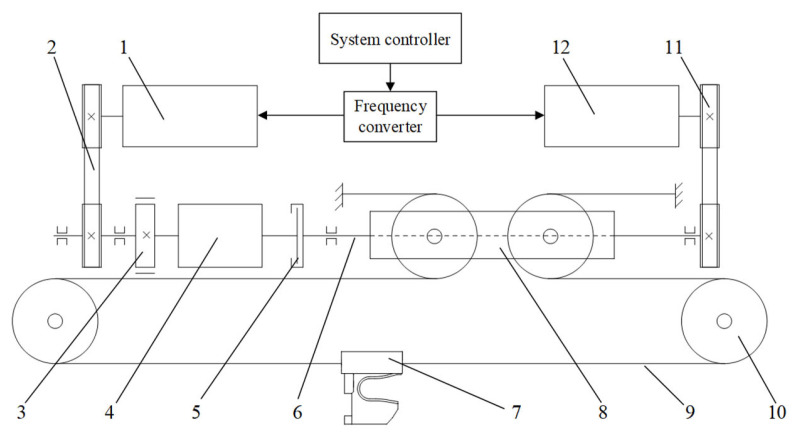
ERSD transmission system schematic diagram. The code names have the following meanings: 1—Towing motor, 2—Towing synchronous belt, 3—Brake, 4—Gearbox, 5—Clutch, 6—Ball screw, 7—Claw, 8—Movable pulley, 9—Chain, 10—Fixed pulley, 11—Capture synchronous belt, 12—Capture motor.

This paper proposes the ERSD transmission system schematic diagram based on the above working characteristics, as shown in Figure 5. The basic functional principle is as follows: when the ERSD performs the towing mission, the towing motor drives the movable pulley through the gearbox and ball screw and then transforms into the transverse motion of the claw through the chain drive mechanism. When the ERSD performs the capture mission, the capture motor directly drives the movable pulley through the ball screw, which is then converted to the transverse motion of the claw through the chain drive mechanism. In the process mentioned above, the working velocity of the two motors is controlled by a frequency converter and system controller. The parameters of the main components of the transmission system are shown in Table 3. The ERSD can also meet relevant performance requirements based on the above calculation results after checking.

### 3.2. Control System Design

**Figure 6:**
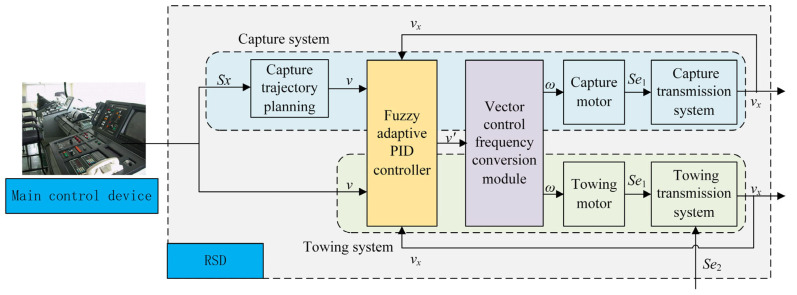
The control system structure.

In this paper, fuzzy adaptive PID control algorithm and vector control frequency conversion algorithm are combined so that the control system can adjust the PID parameters in real time according to the field working state. Moreover, the asynchronous motor and claw would have better velocity tracking performance. The control system structure is shown in Figure 6.

#### 3.2.1. Vector Control Frequency Conversion Module

**Figure 7:**
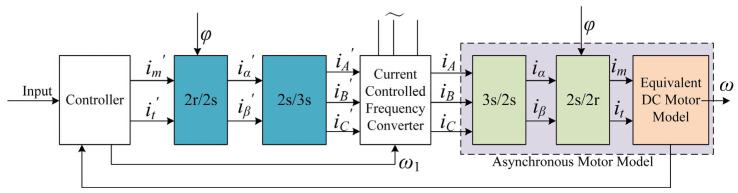
The principle of vector control [22].

As shown in Figure 7, the vector control frequency conversion module equates the current in the three-phase coordinate system of the asynchronous motor to the current in the two-phase static coordinate system. After coordinate transformation, the two-phase stationary coordinate system is equated to the two-phase synchronous rotating coordinate system. If the coordinate system is rotated with the core of the asynchronous motor simultaneously, the DC motor model is obtained. Then, the AC motor can be controlled in the same way as the DC motor [22].

The model of the asynchronous motor in a two-phase synchronous rotating coordinate system is given as follows:

(8) $$\left[\begin{array}{c} u_{sd}\\ u_{sq}\\ u_{rd}\\ u_{rq} \end{array}\right]=\left[\begin{array}{cccc} R_{s}+L_{s}p & -\omega_{1}L_{s} & L_{m}p & -\omega_{1}L_{m}\\ \omega_{1}L_{s} & R_{s}+L_{s}p & \omega_{1}L_{m} & L_{m}p\\ L_{m}p & -\omega_{s}L_{m} & R_{r}+L_{r}p & -\omega_{s}L_{r}\\ \omega_{s}L_{m} & L_{m}p & \omega_{s}L_{r} & R_{r}+L_{r}p \end{array}\right]\left[\begin{array}{c} i_{sd}\\ i_{sq}\\ i_{rd}\\ i_{rq} \end{array}\right],$$

(9) $$\left[\begin{array}{c} \psi_{sd}\\ \psi_{sq}\\ \psi_{rd}\\ \psi_{rq} \end{array}\right]=\left[\begin{array}{cccc} L_{s} & 0 & L_{m} & 0\\ 0 & L_{s} & 0 & L_{m}\\ L_{m} & 0 & L_{r} & 0\\ 0 & L_{m} & 0 & L_{r} \end{array}\right]\left[\begin{array}{c} i_{sd}\\ i_{sq}\\ i_{rd}\\ i_{sq} \end{array}\right],$$

(10) $$T_{e}=n_{p}L_{m}\left(i_{sq}i_{rd}-i_{sd}i_{rq}\right),$$

(11) $$T_{e}-T_{L}=p\omega J/n_{p},$$

(12) $$\omega =\omega_{1}-\omega_{s},$$

where *Ψ_sd_*, *Ψ_sq_*, *Ψ_rd_*, *Ψ_rq_* are stator flux and rotor flux, respectively; *i_sd_*, *i_sq_*, *i_rd_*, *i_rq_*, *u_sd_*, *u_sq_*, *u_rd_*, *u_rq_* are stator current, stator voltage, rotor current, and rotor voltage, respectively; *R_s_*, *R_r_*, *L_s_*, *L_r_*, *L_m_* are stator resistance, stator inductance, rotor resistance, rotor inductance, and stator/rotor mutual inductance; *ω*_1_ is the angular velocity of the coordinate axis d-q relative to the stator; *ω*_s_ is the angular velocity of the coordinate axis d–q relative to the rotor; *ω* is the rotor angular velocity of the asynchronous motor; *n_p_* is the polar logarithm; *T_L_* is the load torque; *J* is the moment of inertia of the motor; *p* is the differential operator.

In Figure 7, if the two-phase rotation coordinate transformation, 2 r/2 s, is offset with the 2 s/2 r transformation inside the asynchronous motor, and the two-phase to three-phase coordinate transformation, 2 s/3 s, is offset with the 3 s/2 s transformation inside the asynchronous motor, then the lag effect of the inverter on the control signal can be ignored and the input control signal can be considered directly equivalent to the output frequency signal. Moreover, if the direction of the *d*-axis along the rotor total flux vector *Ψ_r_* is taken as the *M*-axis, and the *q*-axis rotated 90 degrees counterclockwise, which is perpendicular to the vector *Ψ_r_*, is taken as the *T*-axis, then we get the following equation:

(13) $$\left\{\begin{array}{l} \psi_{rm}=\psi_{r}\\ \psi_{rt}=0 \end{array}\right..$$

Substitute the above equation into the motor model to obtain

(14) $$\left\{\begin{array}{l} \psi_{r}=\frac{L_{m}}{T_{r}p+1}i_{sm}\\ \omega_{s}=\omega_{{}_{1}}-\omega =\frac{L_{m}i_{st}}{T_{r}\psi_{r}}\\ T_{e}=\frac{n_{p}L_{m}}{L_{r}}i_{st}\psi_{r} \end{array}\right..$$

#### 3.2.2. Fuzzy Adaptive PID Controller Design

**Figure 8:**
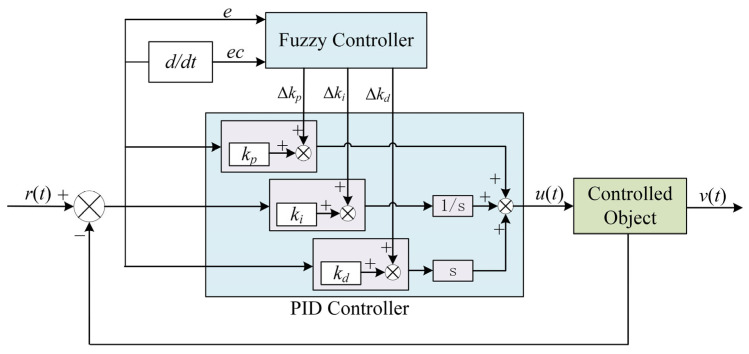
Block diagram of the fuzzy adaptive PID controller [22,23].

The fuzzy adaptive PID controller uses the fuzzy control principle to process the input error and the error change rate and obtain the correction signals of three parameters according to the fuzzy rule table defined by the controller. The correction signals are substituted into Equation (15) to calculate, and the adaptive setting PID parameters are obtained. The controller structure is shown in Figure 8 [22].

(15) $$\left\{\begin{array}{l} k_{p}=k_{p}{}^{\prime }+\Delta k_{p}\\ k_{i}=k_{i}{}^{\prime }+\Delta k_{i}\\ k_{d}=k_{d}{}^{\prime }+\Delta k_{d} \end{array}\right.$$

### 3.3. The Capture Trajectory Planning

According to the above working characteristics of the ERSD, the ERSD requires the velocity curve of the claw to meet the following requirements when performing capture mission:

- The total capture time should be as short as possible, and the maximum capture range should not be less than 2000 mm;
- The velocity changes as smoothly as possible during the capture process;
- When the claw captures the probe rod, the impact force does not exceed 1000 N. (The experimental results show that the allowable capture velocity is approximately 0.8 m/s).

**Figure 9:**
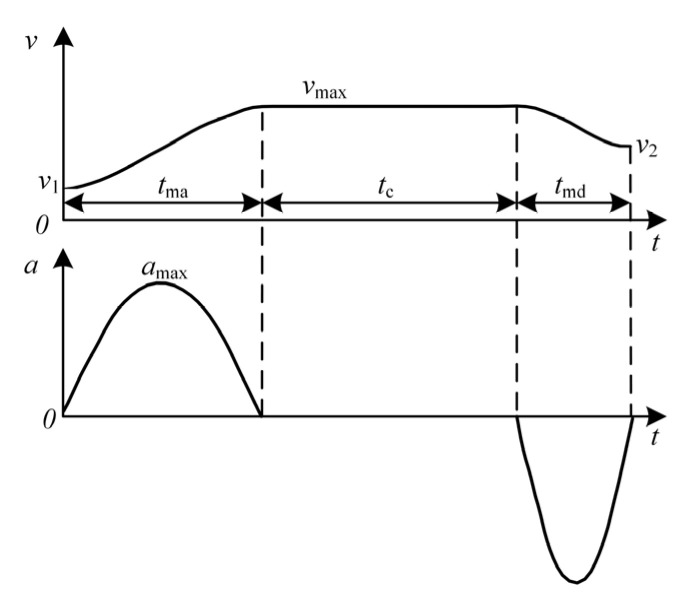
The velocity and acceleration curves.

The capture mission of the ERSD can be described as follows: for a fixed capture range, the claw first starts at a velocity *v*_1_ and accelerates flexibly to a velocity *v*_max_ within a certain period, and then runs at that velocity uniformly for a while. When the claw approaches the probe rod, it enters the deceleration stage and decelerates to *v*_2_ in the remaining distance. At the same time, it reaches the end of the capture range. In this paper, the trigonometric function curve is used to construct the velocity track of the claw [24]. Moreover, the curve is built by taking advantage of the infinite continuous differentiable cosine function, as shown in Figure 9.

The velocity–time function of the addition (decrement) velocity segment in Figure 9 is

(16) $$v(t)=v_{1}+\frac{1}{2}(v_{\max}-v_{1})[1+\cos(\frac{t\pi }{t_{m}}+\pi )],$$

where *t* is the time variable and *t*_m_ is the time required for the acceleration (deceleration) phase.

After simplification, we can obtain

(17) $$v(t)=\frac{1}{2}(v_{\max}+v_{1})-\frac{1}{2}(v_{\max}-v_{1})\cos t^{\prime },$$

where $t^{\prime }=\left(t/t_{m}\right)\pi$ and the velocity curve can be determined by selecting *t*_m_.

When the ERSD captures the helicopter probe rod, the capture range is generally divided into the close range, the mid range, and the far range according to the different positions of the probe rod. The velocity curves of the claws are also adjusted differently for different capture ranges.

1. 1. The far range

When the position of the probe rod in the capture range is *S*_x_ > 1300 mm when the helicopter lands, it is defined as the far range. According to the constructor of Equation (17) above, the trajectory planning curves of each section are as follows.

(18) $$\left\{\begin{array}{ll} v_{a}(t)=\frac{1}{2}(v_{\max}+v_{1})-\frac{1}{2}(v_{\max}-v_{1})\cos{t^{\prime }}_{a} & 0\leq t<t_{m}\\ v_{c}(t)=v_{c} & t_{m}\leq t<t_{m}+t_{c}\\ v_{d}(t)=\frac{1}{2}(v_{\max}+v_{2})-\frac{1}{2}(v_{2}-v_{\max})\cos{t^{\prime }}_{d} & t_{m}+t_{c}\leq t, \end{array}\right.$$

where *v*_c_ and *t*_c_ are the velocity and time of uniform motion; *S*_a_ and *S*_d_ are acceleration distance and deceleration distance; $t_{a}^{\prime }=\left(t/t_{m}\right)\pi$; $t_{d}^{\prime }=\left[\left(t-t_{m}-t_{c}\right)/t_{m}\right]\pi$; $t_{c}=\left(s_{x}-s_{a}-s_{d}\right)/v_{max}$.

The starting velocity is 0.4 m/s, the capture velocity is 0.8 m/s, the maximum velocity is 3.6 m/s, and the acceleration/deceleration time is 0.3 s. The calculated acceleration distance *S*_a_ = 600 mm, deceleration distance *S*_d_ = 660 mm. The minimum capture range is greater than the acceleration and deceleration distance, *S*_x_ > *S*_a_ *+ S*_d_. Moreover, when the probe rod is at the farthest end, the whole capture time is *t* = 2*t*_m_ + *t*_c_ = 0.81 s, which meets the capture time requirement.

2. The mid range

When the position of the probe rod in the capture range is 600 mm < *S*_x_ ≤ 1300 mm when the helicopter lands, it is defined as the mid range. At this time, the capture distance of the claw is relatively short, and the original velocity curve cannot meet the requirements of acceleration and deceleration distance in the minimum remote capture. Therefore, set the starting velocity at 0.4 m/s, the capture velocity at 0.8 m/s, the maximum velocity at 2.4 m/s, and the acceleration/deceleration time at 0.2 s. After calculation, the whole capture time is 0.69 s.

3. The close range

When the position of the probe rod in the capture range is *S*_x_ ≤ 600 mm when the helicopter lands, it is defined as the close range. Because the probe rod is very close to the initial position, the claw does not need to accelerate to a high velocity to complete the capture mission in the specified time. Its velocity curve is Equation (19). Set the starting velocity at 0.4 m/s, capture and maximum at 0.8 m/s, and acceleration/deceleration time at 0.1 s. After calculation, the whole capture time is 0.78 s.

(19) $$\left\{\begin{array}{ll} v_{a}(t)=\frac{1}{2}(v_{2}+v_{1})-\frac{1}{2}(v_{2}-v_{1})\cos t^{\prime } & 0\leq t<t_{m}\\ v_{c}(t)=v_{2} & t_{m}<t \end{array}\right.$$

## 4. System Modeling and Simulation

### 4.1. Dynamic Model of the Transmission System

In this paper, the dynamics model of the ERSD transmission system is established through power bond graph theory [25], in which the energy transfer during the towing mission can be described by the following process:

- The driving torque of the towing motor (*Se*_1_) overcomes the rotational damping (*R*_3_) and drives the active belt wheel to rotate (*I*_2_);
- The active belt wheel drives the driven shaft, electromagnetic brake, and gearbox input shaft to overcome the rotational damping and rotate together (*I*_9_, *R*_10_) through the synchronous belt (TF_1_, TF_2_), during which the length of the synchronous belt will change with the change of the tension force on the belt (*C*_6_);
- After the gearbox (TF_3_), the output shaft drives the clutch and ball screw to rotate together (*I*_13_, *R*_14_);
- The screw transmission pair of the ball screw (TF_4_) transforms the fixed axis rotation of the screw into the axial translation of the movable pulley (*I*_17_, *R*_18_);
- Through pulley transmission (TF_5_), the movable pulley drives the chain and claw to overcome friction damping and move laterally (*I*_23_, *R*_24_); and the length of the chain also varies with the tension (*C*_21_);
- The probe road also applies an external load force to the claw (*Se*_2_).

**Figure 10:**
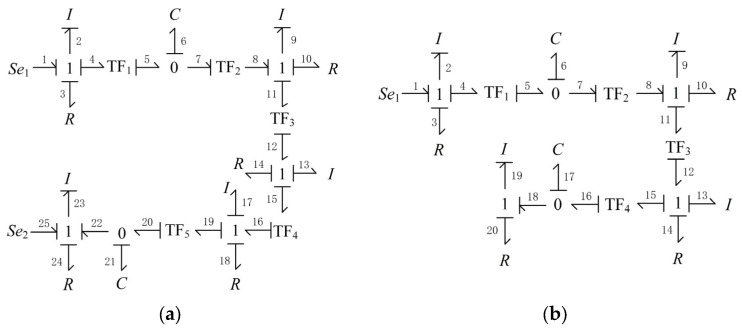
The system bond graph. (**a**) The towing system; (**b**) the capture system.

The system bond graph shown in Figure 10a can be drawn according to the above power flow analysis. Similarly, the capture system model is shown in Figure 10b.

Taking the towing system as an example, the system state equation is expressed as follows:

(20) $${X^{\prime }}_{1}=A_{1}\cdot X_{1}+B_{1}\cdot U_{1}.$$

The generalized momentum *p*_2_, *p*_9_, and *p*_23_ correspond to the inertial elements *I*_2_, *I*_9_, and *I*_23_ with integral causality. The generalized displacement q6 and q21, corresponding to the capacitive elements *C*_6_ and *C*_21_ with integral causality, were selected as the system’s state variables. Its expression is as follows:

(21) $$X_{1}={\left[\begin{array}{ccccc} p_{2} & q_{6} & p_{9} & q_{21} & p_{23} \end{array}\right]}^{T}.$$

The input of the system includes $Se_{1}$ and $Se_{2}$, as shown below:

(22) $$U_{1}={\left[\begin{array}{cc} Se_{1} & Se_{2} \end{array}\right]}^{T}.$$

The relationships between generalized momentum and generalized displacement of energy storage elements *I*_2_, *C*_6_, *I*_9_, *C*_21_, and *I*_23_ are shown as follows:

(23) $$\left\{\begin{array}{l} f_{2}=\frac{1}{I_{2}}p_{2}\\ e_{6}=\frac{1}{C_{6}}q_{6}\\ f_{9}=\frac{1}{I_{9}}p_{9}\\ e_{21}=\frac{1}{C_{21}}q_{21}\\ f_{23}=\frac{1}{I_{23}}p_{23} \end{array}\right..$$

The relationships between potential variables and flow variables of resistive elements *R*_3_, *R*_10_, *R*_14_, *R*_18_, and *R*_24_ are shown as follows:

(24) $$\left\{\begin{array}{l} e_{3}=R_{3}\cdot f_{3}\\ e_{10}=R_{10}\cdot f_{10}\\ e_{14}=R_{14}\cdot f_{14}\\ e_{18}=R_{18}\cdot f_{18}\\ e_{24}=R_{24}\cdot f_{24} \end{array}\right..$$

The relationship between potential variables and flow variables of inertial elements *I*_13_ and *I*_17_ with differential causality is shown as follows:

(25) $$\left\{\begin{array}{l} e_{13}=I_{13}\cdot {f^{\prime }}_{13}\\ e_{17}=I_{17}\cdot {f^{\prime }}_{17} \end{array}\right..$$

The relationships between potential variables and flow variables of converters TF_1_, TF_2_, TF_3_, TF_4_, and TF_5_ are shown as follows:

(26) $$\left\{\begin{array}{c} e_{4}=\frac{1}{m_{1}}\cdot e_{5}\\ f_{5}=\frac{1}{m_{1}}\cdot f_{4} \end{array}\right.,$$

(27) $$\left\{\begin{array}{c} e_{8}=m_{2}\cdot e_{7}\\ f_{7}=m_{2}\cdot f_{8} \end{array}\right.,$$

(28) $$\left\{\begin{array}{c} e_{11}=\frac{1}{m_{3}}\cdot e_{12}\\ f_{12}=\frac{1}{m_{3}}\cdot f_{11} \end{array}\right.,$$

(29) $$\left\{\begin{array}{c} e_{15}=\frac{1}{m_{4}}\cdot e_{16}\\ f_{16}=\frac{1}{m_{4}}\cdot f_{15} \end{array}\right.,$$

(30) $$\left\{\begin{array}{c} e_{19}=\frac{1}{m_{5}}\cdot e_{20}\\ f_{20}=\frac{1}{m_{5}}\cdot f_{19} \end{array}\right..$$

The relationships between potential variables and flow variables of each 0 knot and 1 knot are shown as follows:

(31) $$\left\{\begin{array}{c} e_{2}=Se_{1}-e_{3}-e_{4}\\ f_{1}=f_{2}=f_{3}=f_{4} \end{array}\right.,$$

(32) $$\left\{\begin{array}{c} e_{5}=e_{6}=e_{7}\\ f_{6}=f_{5}-f_{7} \end{array}\right.,$$

(33) $$\left\{\begin{array}{c} e_{9}=e_{8}-e_{10}-e_{11}\\ f_{8}=f_{9}=f_{10}=f_{11} \end{array}\right.,$$

(34) $$\left\{\begin{array}{c} e_{12}=e_{13}+e_{14}+e_{15}\\ f_{12}=f_{13}=f_{14}=f_{15} \end{array}\right.,$$

(35) $$\left\{\begin{array}{c} e_{16}=e_{17}+e_{18}+e_{19}\\ f_{16}=f_{17}=f_{18}=f_{19} \end{array}\right.,$$

(36) $$\left\{\begin{array}{c} e_{20}=e_{21}=e_{22}\\ f_{21}=f_{20}-f_{22} \end{array}\right.,$$

(37) $$\left\{\begin{array}{l} e_{23}=e_{22}+Se_{2}-e_{24}\\ f_{22}=f_{23}=f_{24} \end{array}\right..$$

Combining Equations (23)–(37), the conservation equations of state variables can be obtained in the following form:

(38) $${p^{\prime }}_{2}=Se_{1}-\frac{R_{3}}{I_{2}}p_{2}-\frac{1}{m_{1}C_{6}}q_{6},$$

(39) $${q^{\prime }}_{6}=\frac{1}{m_{1}I_{2}}p_{2}-\frac{m_{2}}{I_{9}}p_{9},$$

(40) $${p^{\prime }}_{9}=\frac{m_{2}m_{3}^{2}m_{4}^{2}I_{9}}{C_{6}\left(m_{3}^{2}m_{4}^{2}I_{9}+m_{4}^{2}I_{13}+I_{17}\right)}q_{6}-\frac{R_{18}+m_{4}^{2}R_{14}+m_{3}^{2}m_{4}^{2}R_{10}}{m_{3}^{2}m_{4}^{2}I_{9}+m_{4}^{2}I_{13}+I_{17}}p_{9}-\frac{m_{3}m_{4}I_{9}}{m_{5}C_{21}(I_{17}+m_{3}^{2}m_{4}^{2}I_{9}+m_{4}^{2}I_{13}}q_{21},$$

(41) $${q^{\prime }}_{21}=\frac{1}{m_{3}m_{4}m_{5}I_{9}}p_{9}-\frac{1}{I_{23}}p_{23},$$

(42) $${p^{\prime }}_{23}=Se_{2}+\frac{1}{C_{21}}q_{21}-\frac{R_{24}}{I_{23}}p_{23}.$$

According to Equations (38)–(42), constant coefficient matrix *A*_1_ and control matrix *B*_1_ can be obtained in the following form:

(43) $$A_{1}=\left[\begin{array}{ccccc} -\frac{R_{3}}{I_{2}} & -\frac{1}{m_{1}C_{6}} & 0 & 0 & 0\\ \frac{1}{m_{1}I_{2}} & 0 & -\frac{m_{2}}{I_{9}} & 0 & 0\\ 0 & \frac{m_{2}m_{3}^{2}m_{4}^{2}I_{9}}{C_{6}\left(m_{3}^{2}m_{4}^{2}I_{9}+m_{4}^{2}I_{13}+I_{17}\right)} & -\frac{R_{18}+m_{4}^{2}R_{14}+m_{3}^{2}m_{4}^{2}R_{10}}{m_{3}^{2}m_{4}^{2}I_{9}+m_{4}^{2}I_{13}+I_{17}} & -\frac{m_{3}m_{4}I_{9}}{m_{5}C_{21}(I_{17}+m_{3}^{2}m_{4}^{2}I_{9}+m_{4}^{2}I_{13}} & 0\\ 0 & 0 & \frac{1}{m_{3}m_{4}m_{5}I_{9}} & 0 & -\frac{1}{I_{23}}\\ 0 & 0 & 0 & \frac{1}{C_{21}} & -\frac{R_{24}}{I_{23}} \end{array}\right],$$

(44) $$B_{1}=\left[\begin{array}{cc} 1 & 0\\ 0 & 0\\ 0 & 0\\ 0 & 0\\ 0 & 1 \end{array}\right].$$

Similarly, the capture system state equation is shown as follows:

(45) $${X^{\prime }}_{2}=A_{2}\cdot X_{2}+B_{2}\cdot U_{2}.$$

The system state variables are expressed as follows:

(46) $$X_{2}={\left[\begin{array}{ccccc} p_{2} & q_{6} & p_{9} & q_{17} & p_{19} \end{array}\right]}^{T}.$$

The input of the system is only the output torque of the motor spindle $Se_{1}$, as shown below

(47) $$U_{2}=\left[Se_{1}\right].$$

Moreover, the constant coefficient matrix *A*_1_ and control matrix *B*_1_ can be obtained in the following form:

(48) $$A_{2}=\left[\begin{array}{ccccc} -\frac{R_{3}}{I_{2}} & -\frac{1}{m_{1}C_{6}} & 0 & 0 & 0\\ \frac{1}{m_{1}I_{2}} & 0 & -\frac{m_{2}}{I_{9}} & 0 & 0\\ 0 & \frac{m_{2}m_{3}^{2}I_{9}}{C_{6}\left(m_{3}^{2}I_{9}+I_{13}\right)} & -\frac{m_{3}^{2}R_{10}+R_{14}}{m_{3}^{2}I_{9}+I_{13}} & -\frac{m_{3}I_{9}}{m_{4}C_{17}(m_{3}^{2}I_{9}+I_{13})} & 0\\ 0 & 0 & \frac{1}{m_{3}m_{4}I_{9}} & 0 & -\frac{1}{I_{19}}\\ 0 & 0 & 0 & \frac{1}{C_{17}} & -\frac{R_{20}}{I_{19}} \end{array}\right],$$

(49) $$B_{2}={\left[\begin{array}{ccccc} 1 & 0 & 0 & 0 & 0 \end{array}\right]}^{T}.$$

### 4.2. System Controllability Analysis

The discriminant matrix *C*_d_ is introduced to judge the system controllability, as shown below:

(50) $$C_{d}=\left[\begin{array}{ccccc} B & AB & A^{2}B & \cdots & A^{n-1}B \end{array}\right],$$

where *n* is the dimension of state variable *X*. When the rank of *C*_d_ is equal to *n*, the system is manageable.

According to Table 4 and Table 5, calculate the *A*_1_, *B*_1_, *A*_2_, and *B*_2_ values and substitute them into Equation (50). It can be concluded that rank (*C*_d1_) = 5 and rank (*C*_d2_) = 5, which indicates that the system can be controlled during the towing and capture mission.

### 4.3. System Simulating

The capture system simulation model and traction system simulation model were built to verify the feasibility of the ERSD transmission scheme and control system.

1. Capture system simulation

**Figure 11:**
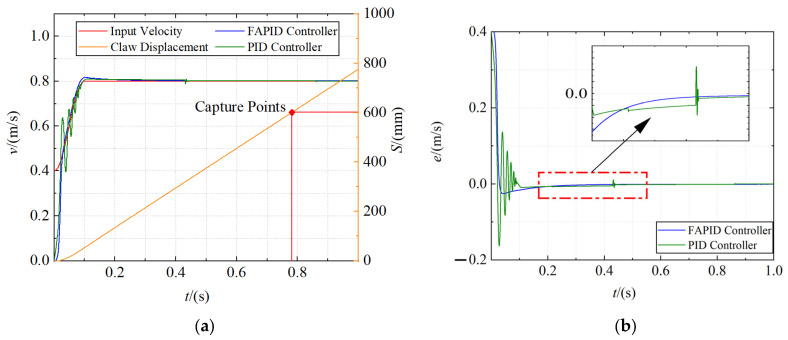
The capture curve of the close range (600 mm). (**a**) The velocity curve and displacement curve; (**b**) the error curve.

**Figure 12:**
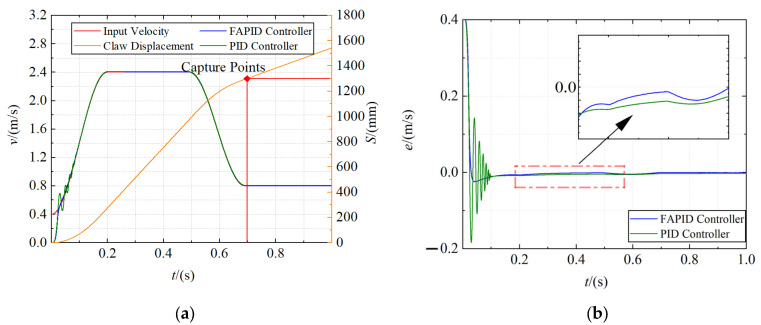
The capture curve of the mid range (1300 mm). (**a**) The velocity curve and displacement curve; (**b**) the error curve.

**Figure 13:**
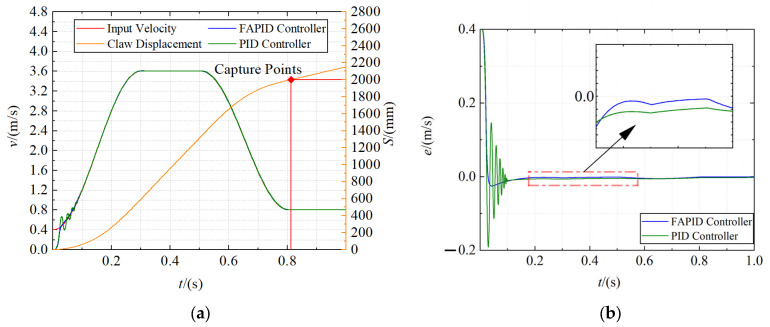
The capture curve of the far range (2000 mm). (**a**) The velocity curve and displacement curve; (**b**) the error curve.

The positions of the probe road were set as 600 mm, 1300 mm, and 2000 mm, respectively, and the capture velocity curve was generated through the velocity trajectory planning module. Fuzzy adaptive PID controller and traditional PID controller are used as velocity controllers for simulation, respectively. The dynamics model parameters used in the simulation are shown in Table 4. The total simulation time was set to 1 s, and the acquisition velocity curve, displacement curve, and error curve of claw were obtained, as shown in Figure 11, Figure 12 and Figure 13, respectively.

It is obvious from the figures that the claw can move along the input velocity curve, and the maximum capture time of the close and far range is approximately 0.8 s, and the mid range is even reduced to 0.7 s. In addition, the fuzzy adaptive PID controller has an obvious control effect in the motor acceleration stage, which makes the overshoot smaller and the acceleration more stable. However, the traditional PID controller has a violent shock in the motor acceleration stage and usually reaches a steady state after the motor starts at 0.1 s. Moreover, the tracking error of the fuzzy adaptive PID controller is smaller in the constant speed stage.

2. Towing system simulation

**Figure 14:**
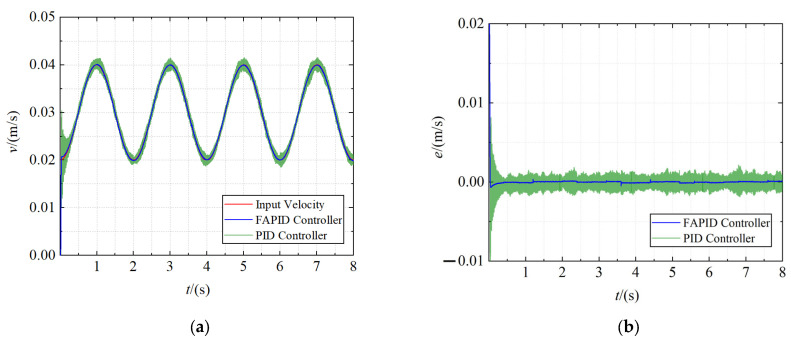
The towing curve. (**a**) The velocity tracking curve; (**b**) the error curve.

The given traction velocity is a sinusoidal curve with a maximum of 0.04 m/s, minimum of 0.02 m/s, and period of 2 s. Meanwhile, with the help of the Uniform Random Number tool, the maximum Random interference load of 34,000 N is imposed. The dynamics model parameters used in the simulation are shown in Table 5. Set the total simulation time to 8 s. The velocity tracking curve and the error curve of the claw are obtained, as shown in Figure 14.

As shown in the figures, when a high random load is applied to the claw, the traditional PID controller is difficult to achieve a steady state in the motor acceleration stage and high-frequency clutter appears in the speed tracking stage. In contrast, the speed curve obtained using the fuzzy adaptive PID controller has good anti-interference ability, excellent dynamic response characteristics, and steady-state characteristics. It can meet the requirements of the ERSD when towing helicopters under high loads.

## 5. Discussion

**Figure 15:**
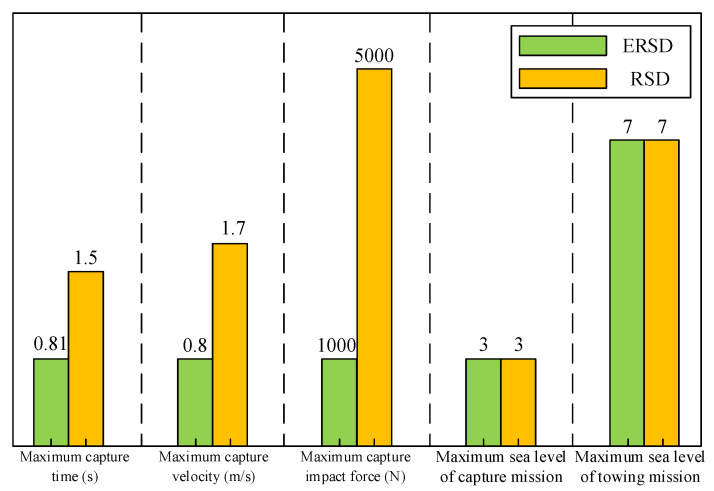
Comparison of operating parameters between RSD and ERSD.

In this paper, the working characteristics of the RSD are analyzed, and the implementation scheme of the ERSD transmission system is proposed. The capture velocity trajectory of the claw is designed, and the control system is created by combining vector control frequency conversion algorithm and fuzzy adaptive PID control algorithm. On this basis, the dynamics model of the transmission system of ERSD was built through power bond graph theory, and the controllability of the system was analyzed. The simulation test was carried out in MATLAB/Simulink. It can be seen from the simulation results that the ERSD proposed in this paper has the following significant characteristics:

1. As shown in Figure 15, based on meeting the functional requirements of the original RSD, the ERSD reduces the maximum capture time by approximately 47%, the maximum capture velocity by approximately 53%, and the capture impact force by approximately 80%;
2. The claw can still have good velocity tracking performance under the maximum interference load of 34,000 N.

The ERSD proposed in this paper enables ASIST to be compatible with small shipborne aircraft equipped with helicopter-like landing systems such as the UAV and improves the efficiency of ASIST towing operations. The research results obtained in this paper provide an essential basis for the subsequent production of ERSD and the design of an ASIST-based automatic traction system.

## Figures and Tables

**Table 1:** Parameters of the shipborne helicopter.

Name of Parameters	Value	Name of Parameters	Value
*L*_H_	0.9 m	*M*_H_	10,000 kg
*L*_E_	0.9 m	*L*	7 m
*B*	3.5 m	*H*	2 m
*L*_W_	0.9 m	*H*_W_	1.3 m

**Table 2:** Sea conditions table of the ship.

Name of Parameters	3-Level Sea Condition	7-Level Sea Condition
*ρ*	1.29 kg/m^3^	1.29 kg/m^3^
*θ*	10°	16°
*a*_x_	1 m/s^2^	2 m/s^2^
*a*_z_	2 m/s^2^	4 m/s^2^
*V*_W_	12 m/s	30 m/s

**Table 3:** The parameters of the transmission system.

Name of Parameters	Value	Name of Parameters	Value
Power of the asynchronous motor	5 kW	Power of frequency converter	5.5 kW
The reduction ratio of the gearbox	40	The lead of the ball screw	20 mm
Friction torque of the brake	30 N·m	Capture area width	2000 mm

**Table 4:** Parameters of the towing system.

Name of Parameters	Value	Name of Parameters	Value
*I*_2_	1.04 × 10^−3^ kg·m^2^	*R*_14_	2.5 × 10^−3^ N·m/(rad/s)
*R*_3_	8.2 × 10^−4^ N·m/(rad/s)	TF_4_(m_4_)	2π/(20 × 10^−3^) rad/m
TF_1_(m_1_)	1/48.51 × 10^−3^ rad/m	*I*_17_	35.753 kg
*C*_6_	1/15,200 m/N	*R*_18_	40 N/(m/s)
TF_2_(m_2_)	48.51 × 10^−3^ m/rad	TF_5_(m_5_)	1/2
*I*_9_	3.865 × 10^−3^ kg·m^2^	*C*_21_	0.5 × 10^−7^ m/N
*R*_10_	3.8 × 10^−3^ N·m/(rad/s)	*I*_23_	10,044.328 kg
TF_3_(m_3_)	40	*R*_24_	30 N/(m/s)
*I*_13_	2.295 × 10^−2^ kg·m^2^	-	-

**Table 5:** Parameters of the capture system.

Name of Parameters	Value	Name of Parameters	Value
*I*_2_	1.04 × 10^−3^ kg·m^2^	TF_3_(m_3_)	2π/(20 × 10^−3^) rad/m
*R*_3_	8.2 × 10^−4^ N·m/(rad/s)	*I*_13_	35.753 kg
TF_1_(m_1_)	1/48.51 × 10^−3^ rad/m	*R*_14_	40 N/(m/s)
*C*_6_	1/15,200 m/N	TF_4_(m_4_)	1/2
TF_2_(m_2_)	48.51 × 10^−3^ m/rad	*C*_17_	0.5 × 10^−7^ m/N
*I*_9_	9.22 × 10^−3^ kg·m^2^	*I*_19_	44.328 kg
*R*_10_	2 × 10^−3^ N·m/(rad/s)	*R*_20_	30 N/(m/s)
